# Case Report: Double trabecular metal cup without auxiliary screws for severe acetabular bone defects

**DOI:** 10.3389/fsurg.2025.1655804

**Published:** 2025-08-22

**Authors:** Ming Xia, Dongbo Li, Chunquan Zhu, Lihui Sun, Dongsong Li

**Affiliations:** Department of Orthopedics, The First Hospital of Jilin University, Changchun, Jilin, China

**Keywords:** acetabular bone defects, revision total hip arthroplasty, double-cup technique, screw fixation, case report

## Abstract

**Background:**

Acetabular reconstruction is often challenging in revision hip arthroplasty, especially in the face of moderate to severe acetabular bone deficiency. In some severe bone defects, double-metal tantalum cups can improve the contact area between bone and implants, increase the surface area for bone ingrowth, and better restore the anatomical position of the acetabulum. Furthermore, with a good press-fit, the auxiliary screw has a minimal effect on acetabular cup stability.

**Case presentation:**

We report a case of a 63-year-old male patient who was diagnosed with loosening prosthesis after total hip arthroplasty and whose preoperative radiograph suggested a large bone defect in the acetabulum. Due to the large amount of purulent fluid found in the joint cavity during the first revision surgery, a decision was made to stage-1spacer placement followed by a second-stage revision. In the second-stage revision, we utilized a double-cup technique to fill the large acetabular bone defect.

**Conclusions:**

In the revision total hip arthroplasty, if the acetabular bone defect is severe, a double-metal tantalum cup structure can be used to reconstruct the acetabular structure, restore the center of rotation of the hip joint. Under good press-fit conditions, the metal tantalum cup can obtain initial stability of sufficient strength even without screw fixation, and achieve secondary stability through bone growth.

## Introduction

With the increase in the total hip arthroplasty (THA) operation volume, the prolongation of patient life, and the trend of younger surgery, the number and complexity of revision surgery have also increased correspondingly. In revision total hip arthroplasty, there are a number of treatment options for massive acetabular defects. These include reconstruction with jumbo acetabular components, massive structural allografts, bone impaction grafting, oblong cups, antiprotrusio cages, custom triflanged acetabular components, trabecular metal (Zimmer) augments, and shells (1). Structural allografts and antiprotrusio cages have good therapeutic effects. However, aseptic loosening and allograft resorption or collapse are still the reasons for long-term failure (2). Bone impaction grafting is suitable for simple cavity defects, but there is a high risk of hip joint failure in Paprosky type IIIb type defects (3). Triflanged acetabular components are costly, surgery is typically delayed 4–6 weeks before implant fabrication, surgical insertion requires an expandable surgical approach, and there is a risk of superior gluteal nerve injury (4). Oblong bone cups may have a limited role in the management of specific bone loss patterns in revision total hip arthroplasty; however, their use has largely been superseded by modular porous metal augments (5). Previous studies have shown that modular reconstruction systems based on tantalum metal cups, eventually associated with tantalum metal augments, were increasingly used in acetabular revision surgery (6). In Paprosky type IIIA and IIIB defects, however, the acetabular defect can be quite large, making the use of augments cumbersome, if not impossible, in some cases. In addition, depending on the size and shape of certain defects, it may be difficult to locate and attach metal augments to optimize surface contact and achieve biological growth (7).

The “double trabecular metal cup” construct described herein differs from traditional augment-based reconstruction in three principal ways. First, instead of multiple small augments, two hemispherical cups are press-fitted concentrically, thereby converting an irregular defect into two geometrically predictable spherical cavities. Second, the outer cup acts simultaneously as an augment and as a scaffold for the inner bearing cup, eliminating the need for additional screw fixation when adequate press-fit is achieved. Third, by cementing the cups together as a monoblock, micromotion between components is minimized—a theoretical advantage over multi-piece augment constructs.

Although prior studies have reported encouraging short-term outcomes with the double-cup technique, they either included heterogeneous defect types or routinely used screw supplementation (8, 9). In this case, we used a “double-cup” structure with a metal tantalum cup as an augment, which can increase the surface area of bone ingrowth and restore the anatomical position and support ability of the hip joint. Preoperative computed tomography (CT)-based spatial modeling of the innominate bone allows accurate quantification of bone loss and virtual fitting of trial components, thereby reducing intraoperative guesswork (10). Three-dimensional (3D) printing has been successfully applied to produce life-size anatomical replicas for surgical rehearsal, patient-specific cutting guides, and even customized porous implants (11, 12). In the present case, a 1:1 stereolithographic model derived from the patient's pelvic CT data was printed preoperatively to simulate cup positioning and to verify that the planned double-cup construct could be seated on three residual bony struts (ilium, ischium, pubis) without screw fixation. This surgical strategy significantly reduces the cranial migration of the hip center of rotation (COR), restores its anatomical position, and improves the function of the abductors.

Primary fixation of cementless cups is critical in achieving a mechanical environment for secondary fixation through bone ingrowth. The press-fit technique is extremely effective in obtaining adequate initial stability of the acetabular cup, so that good press-fit will result in satisfactory stability and the auxiliary screw will have little effect on whole-cup stability with a good press-fit (13, 14). When proper press-fitting cannot be achieved, the stability of the acetabular prosthesis is greatly reduced, and screws are required to reinforce the stability of the cup (13).

## Case report

### History and preoperative preparation

**Figure 1:**
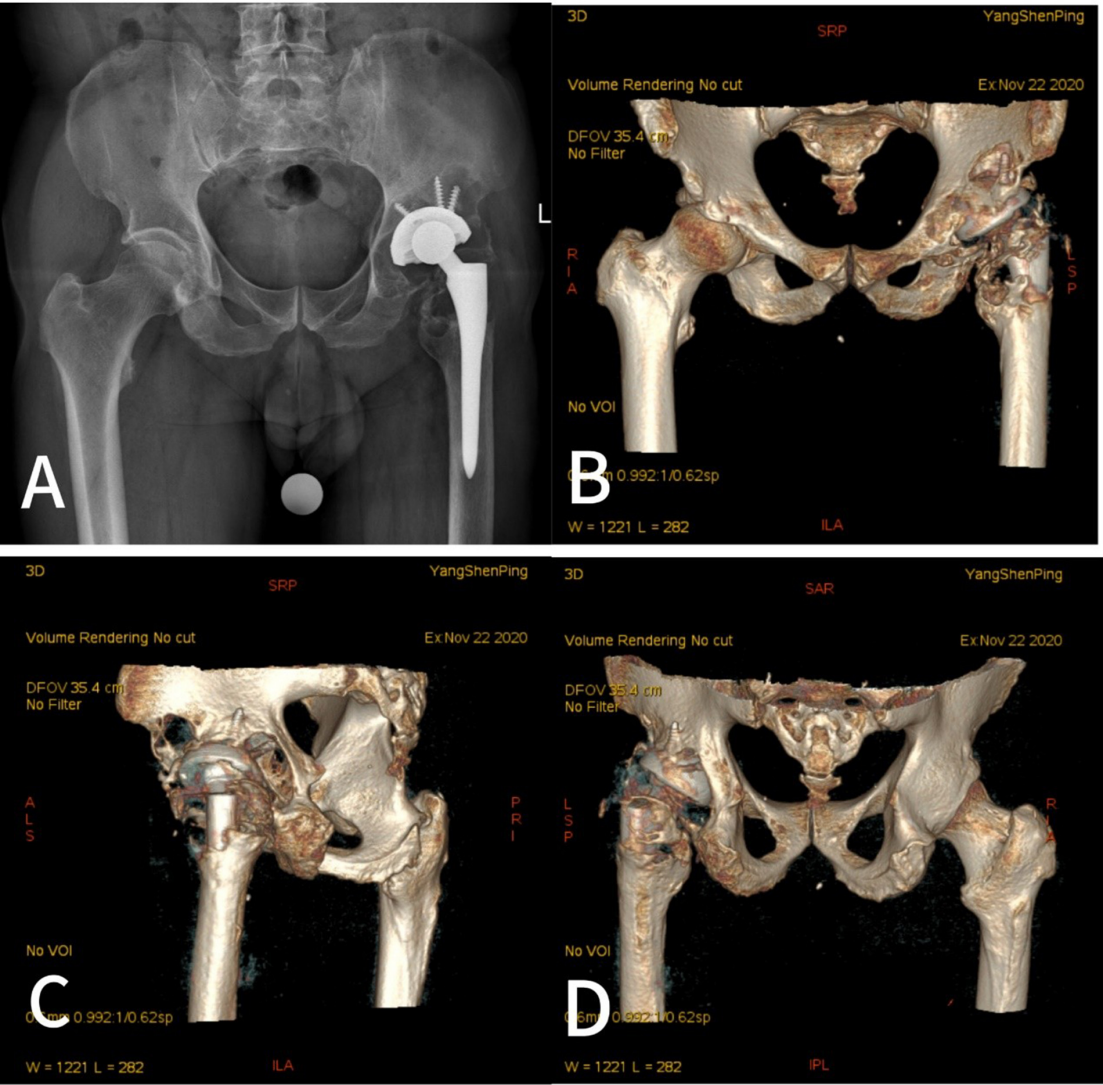
Preoperative imaging showed massive medial and superior acetabular bone loss and proximal displacement of the center of rotation. **(A)** Preoperative radiograph. **(B–D)** Computed tomography and three-dimensional reconstructions.

A 63-year-old male patient underwent left total hip arthroplasty due to traumatic osteoarthritis in 2009. In November 2020, he consciously felt pain in the left groin and was diagnosed at our hospital with prosthetic loosening after left total hip arthroplasty. Plain radiography of the left hip showed massive bone destruction in the acetabulum, and medial and vertical deviation of the center of rotation (Figure 1A). CT and 3D reconstructions indicated serious bone destruction. There was a large defect in the posterior wall of the acetabulum, osteolysis of the anterior lower wall of the iliac bone resulting in a transmural bone defect, a severe bone defect of the pubic bone branch, and a complete loss of the structure of the acetabular ring, which was consistent with the characteristics of Paprosky type IIIB (Figures 1B–D). Chemical examination report showed that white blood cells, erythrocyte sedimentation rate, and C-reactive protein (CRP) were all normal. Physical examination revealed a positive Trendelenburg sign, 2 cm limb-length discrepancy, painful passive hip flexion limited to 70°, and audible crepitus on rotation. The absence of fever or systemic signs argued against acute sepsis; nevertheless, the combination of progressive groin pain and large effusion on imaging necessitated exclusion of periprosthetic joint infection (PJI) and aseptic loosening. Differential diagnoses considered were chronic late PJI, aseptic loosening with secondary synovitis, and metallosis-related osteolysis.

**Figure 2:**
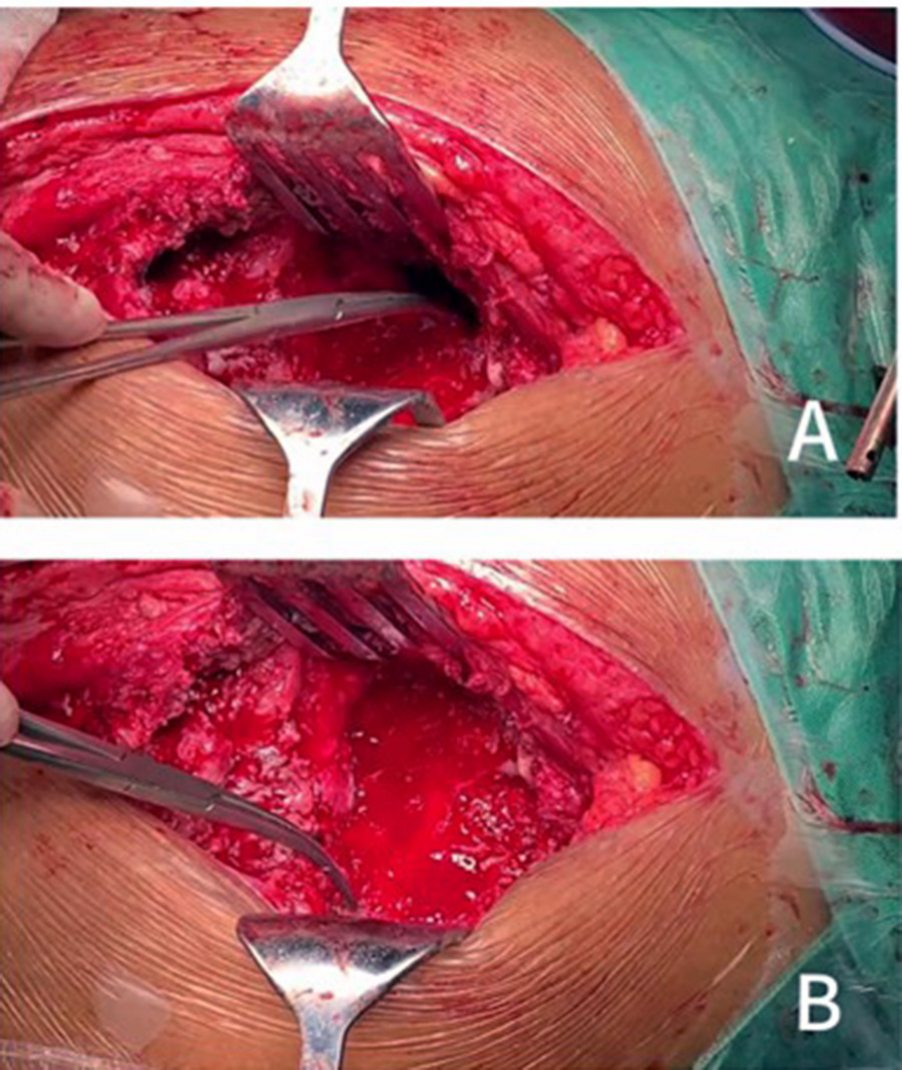
Intraoperatively, there was a large defect in the posterior wall of the acetabulum, osteolysis of the anterior inferior wall of the ilium leading to a transmural bone defect **(A)**, and a severe bone defect in the pubic symphysis **(B)**.

**Figure 3:**
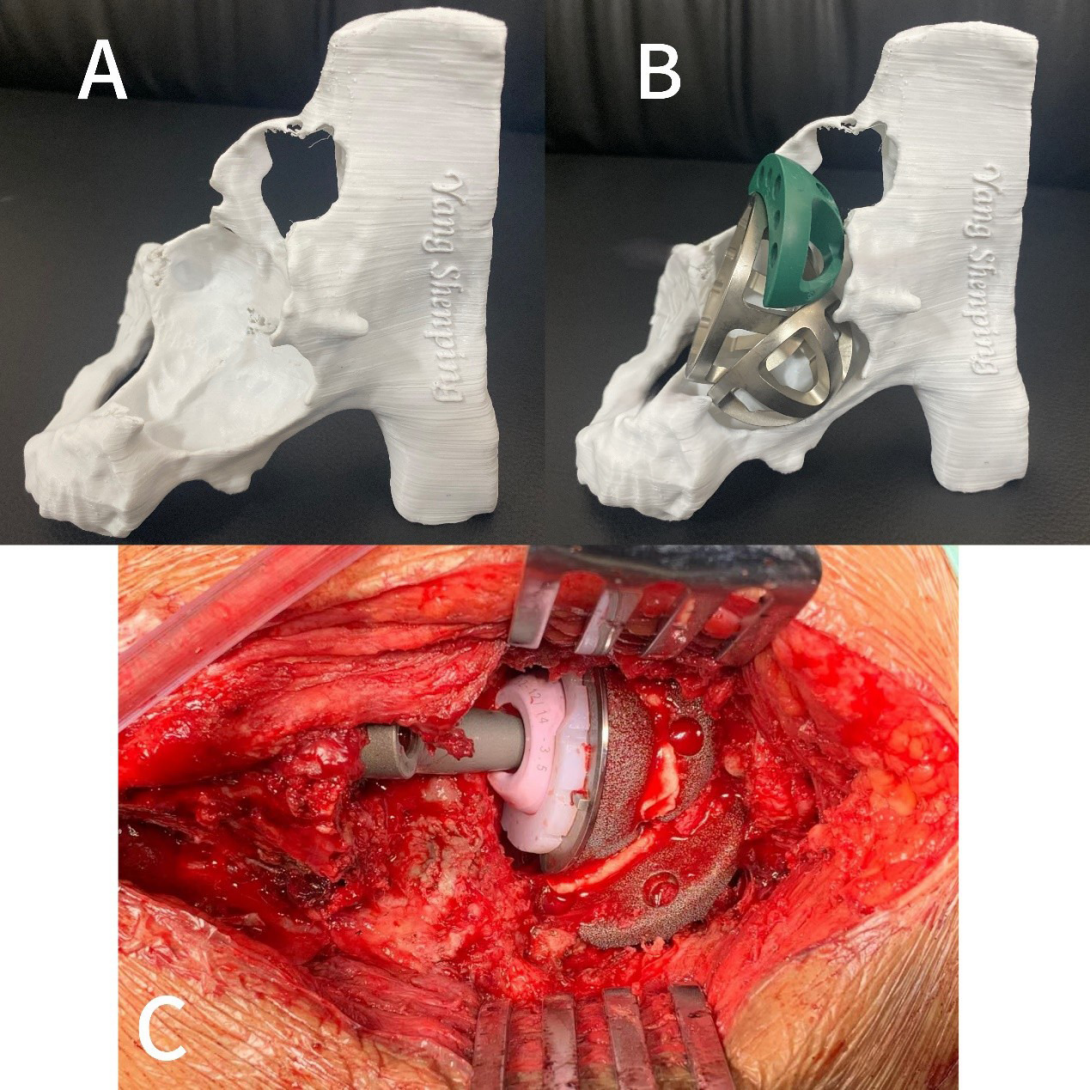
**(A,B)** Preoperative 3D printing and planning. **(C)** Intraoperative application of the double-cup construct to reconstruct acetabular bone defects.

On 8 December 2020, a revision was performed on the total hip arthroplasty. During the operation, it was found that the patient had greater bone defects (Figure 2) and a large volume of purulent fluid in the joint cavity. Purulent fluid was extracted for examination, with the pathological findings showing obvious purulent cells, indicating acute infection. Because the Masquelet-type bone loss (combined medial and superior wall deficiency) precluded single-stage reconstruction, a two-stage protocol was adopted: (1) thorough debridement, modular antibiotic-loaded cement spacer placement, and microbiological sampling; and (2) definitive reconstruction once inflammatory markers normalized and repeat cultures were negative. A stereolithographic pelvic model, produced from preoperative CT data and 3D reconstructions, confirmed a severe acetabular defect with insufficient global bone stock for conventional cup fixation; however, three distinct residual support pillars were identified on the ilium, ischium, and pubis (Figures 3A,B). Therefore, the stability of the acetabulum can be reconstructed based on these three support points to restore medial and vertical deviation. The idea of acetabular reconstruction with double tantalum cups was generated to bring the center of rotation of the hip close to the normal anatomical center and to restore the length and the normal movement of the lower extremity. Another revision of the left total hip arthroplasty was planned on 20 January 2021.

### Surgical procedure

The patient was operated on under anesthesia with a posterolateral approach to the hip joint and full exposure of the acetabulum. Hemispheric reamers were used to reach a well-vascularized bone bed, and attention should be paid to avoiding the aggravation of acetabular defects and to preserving the residual structure of the acetabulum. One acetabular cup was placed on the host bone for fixation with the support points of the ilium and ischium bones, which creates a support point for the fixation of the second tantalum cup, with the dual function of filling the bone gap and supporting the initial fixation of the acetabular fossa; and the other was fixed with the support point of the pubis. Then, a tantalum block was used to compress and fill the acetabulum defect to firmly fix the two acetabulum cups on the severe acetabulum defect of the host bone, and maximize the contact between the entire structure and the remaining pelvic bone. The three implants were adequately cemented and fixed to each other with bone cement to avoid micro-movement and wear between the cups to form a single prosthesis (Figure 3C). A modular titanium-tapered femoral stem and cobalt-chromium femoral head were implanted, and the procedure was completed uneventfully. The hip joint has good movement in all directions, and intraoperative radiographs show that the hip joint prosthesis is in a good position.

No intraoperative adverse events were recorded: specifically, no neuro-vascular injury, iatrogenic fracture, or unanticipated blood loss (>650 mL anticipated). Postoperative surveillance (in-hospital and at 6 weeks, 3 months, 6 months, 12 months, and 22 months) identified the following: wound healing: primary closure intact, no dehiscence or infection; thrombo-embolic events: duplex ultrasound negative at discharge and at 6 weeks; dislocation: nil episodes; and radiological adverse findings: no progressive radiolucent lines, osteolysis, or cup migration. Thus, the Clavien–Dindo classification remained at grade 0 throughout the observation period. The surgical and implant parameters were summarized in Table 1.

**Table 1:** Surgical and implant parameters.

Parameter	Value
Cup and stem details	
Outer tantalum cup (augment)	58 mm, Trabecular Metal™ (Zimmer-Biomet)
Inner tantalum cup (bearing)	54 mm, Trabecular Metal™ (Zimmer-Biomet)
Cement mantle	2–3 mm, Palacos® R + G (PMMA + gentamicin)
Femoral stem	130 mm modular titanium-tapered, HA-coated
Inclination (radiographic)	43° (target 45° ± 5°)
Anteversion (radiographic)	18° (target 15° ± 5°)
Operative data	
Operative time (2nd stage)	210 min
Estimated blood loss	650 mL
Laboratory/microbiology	
1st stage (December 2020)	
WBC	7.12 × 10^9^ L^−1^
ESR	8.45 mm h^−1^
CRP	3.56 mg L^−1^
Cultures	No growth (aerobic/anaerobic/fungal, 5 days)
2nd stage (January 2021)	
WBC	5.07 × 10^9^ L^−1^
ESR	4.39 mm h^−1^
CRP	0.79 mg L^−1^
Cultures	All negative (7 days)
Complications	
Intraoperative	None
Postoperative (22 months)	None

CRP, C-reactive protein; ESR, erythrocyte sedimentation rate; PMMA, polymethylmethacrylate; WBC, white blood cell.

### Follow-up

**Figure 4:**
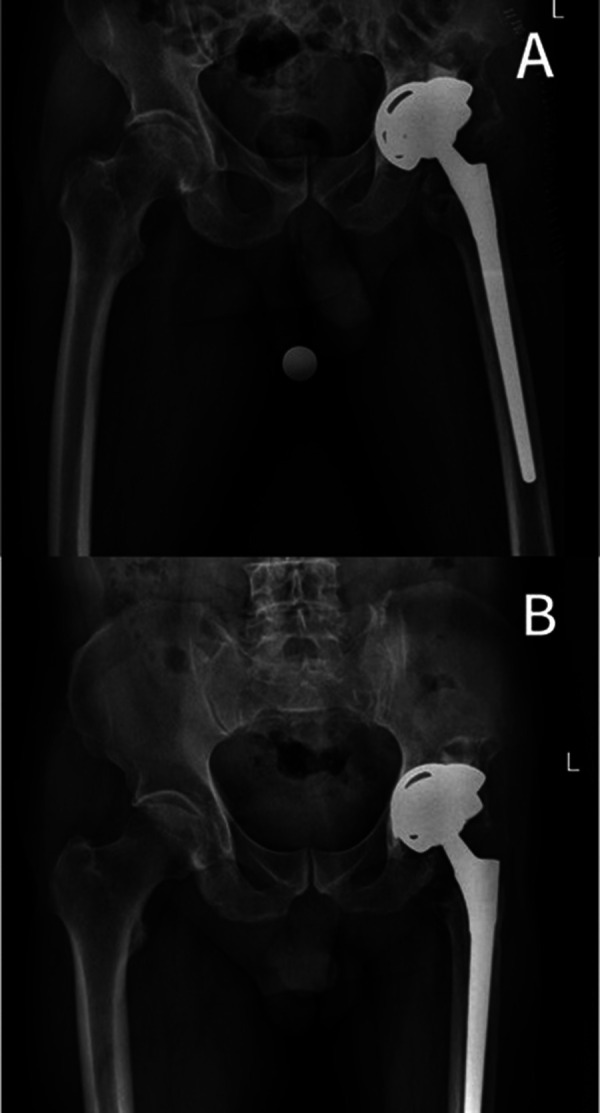
**(A)** Postoperative radiographs. **(B)** Radiographs at the last follow-up.

The postoperative radiograph results were satisfactory, and the acetabular position was accurate (Figure 4A). At 6 weeks postoperatively, the patient began to attempt weightbearing, and after 3 months, the weightbearing gradually increased. At the last follow-up, 22 months postoperatively, the patient was able to move without any support or hip pain. The preoperative Harris Hip Score was 39 and improved at the latest follow-up, with a score of 78. Examination showed that the patient had a good range of motion of the hip joint and no pain in the hip joint with passive motion. Review radiographs showed no evidence of loosening or migration, and the prosthesis remained stable (Figure 4B). The overall chronological timeline of the case was summarized in Table 2.

**Table 2:** Chronological timeline.

Date (yyyy-mm-dd)	Event
2009-03-15	Primary left total hip arthroplasty for post-traumatic osteoarthritis
2020-11-10	Onset of progressive left groin pain; radiographs show acetabular osteolysis
2020-11-20	CT/3D reconstruction confirms Paprosky type IIIB defect; inflammatory markers within normal limits.
2020-12-08	First-stage revision: debridement, removal of loose components, insertion of antibiotic-loaded cement spacer; purulent fluid samples obtained
2020-12-09 to 2021-01-12	Intravenous antibiotics (vancomycin + ceftazidime) guided by microbiology; repeated serum CRP/ESR normalization
2021-01-05	Multidisciplinary board confirms eradication of infection; second-stage surgery scheduled.
2021-01-20	Second-stage reconstruction: double trabecular metal cup construct without screws; operative time 210 min; EBL 650 mL
2021-01-20 to 2021-02-02	Inpatient recovery; DVT prophylaxis with low-molecular-weight heparin; standardized pain and physiotherapy protocols
2021-03-08	6-week radiographs: satisfactory cup position, no migration
2021-04-20	Gradual full weightbearing commenced.
2022-11-18	22-month follow-up: no pain, no walking aids, HHS improved from 39 to 78

CT, computed tomography; DVT, deep vein thrombosis; EBL, estimated blood loss; HHS, Harris Hip Score.

## Discussion

In severe acetabular bone defects, maximizing the stability and intimate contact of the host bone with the acetabular prosthesis is essential to obtain osseointegration and a successful prognosis. In the double tantalum cups structure, a porous tantalum cup is used as an augment to fill the large acetabular bone defect, to obtain primary stability using a press-fit technique and screws, and to create a partial anchoring area for the lateral tantalum cup. Due to the large surface area in contact with the host bone, the secondary stability of the implant is achieved through bone ingrowth. The second tantalum cup is placed at the proper angle and position using a press-fit technique, and the two tantalum cups are integrated with bone cement. This technique allows the two main problems in hip revision surgery to be addressed separately: bone loss and stability (8). In addition, it helps restore horizontal and vertical offset and helps to bring the center of rotation of the hip joint to its anatomical position.

Webb et al. (7) reported the short-term results of 20 cases of Paprosky type III defects treated by the double-cup technique. At an average follow-up of 2.4 years, they reported a 100% survival rate for aseptic loosening and an 80% survival rate for revision for any reason. Loppini et al. (9) reported 16 patients with Paprosky type III defect undergoing acetabular revision, of which only 1 (6.3%) had a non-progressing translucent line around the acetabulum. No patient underwent acetabular component revision surgery for any reason. Chiarlone et al. reported nine patients with double-cup structures with a mean follow-up of 35.3 ± 10.8 months and a 100% survival rate for aseptic loosening and 88.9% for revision of any cause (8). Zhang et al. reported 18 patients with Paprosky type III acetabular defects who underwent revision surgery using double trabecular metal cups, with a median follow-up of 61.0 months, and no patients underwent re-revision due to loosening or any other reason (15).

In addition, adequate initial fixation of the acetabular prosthesis in hip surgery is necessary to achieve long-term survival. The initial stability of the acetabular cup mainly depends on press-fitting, and with a good press-fit, the auxiliary screw has little effect on the stability of the cup. Ni et al. pointed out in a meta-analysis that there was no statistically significant difference in migration or loosening between THAs with and without screws, as well as in the rate of reoperation or revision between the two surgical methods (16). In addition, Ni et al. also published a systematic review stating that acetabular cups can achieve sufficient stability even without screws under press-fit fixation, and there is no difference in many results between acetabular cups without screws and those with screws (17). An *in vitro* biomechanical study showed that the acetabular cup can be satisfactorily stabilized with a simple press-fit without the use of screws, and that auxiliary screws have little effect on the stability of the acetabular cup (13). Roth et al. (18) compared 101 cementless cups implanted by press-fit fixation without the use of screws and 110 cementless cups with additional fixation with one to three screws at the upper part of the acetabulum. They concluded that if good press-fit fixation is achieved, additional screw fixation is not important because the cementless cup with only press-fit reduces radiographic changes around the cup and does not have any clinical disadvantage.

Unlike jumbo cups, which require ≥50% host-bone contact and are therefore unsuitable when the acetabular rim is absent, the double-cup construct fashions its contact surface by interposing an outer trabecular metal “cup-augment” (19). Compared to structural allografts, our approach eliminates the risks of late collapse and disease transmission, but sacrifices the biological remodeling potential of fresh graft (20). Modular tantalum augments offer greater flexibility in small or focal defects; however, in the global Paprosky type IIIB defect presented here, multiple augments would have been required, increasing construct complexity, operative time, and the number of potential micromotion interfaces.

Operative time in our case was 210 min—largely attributable to trial reductions and 3D model referencing. Cost analysis revealed that two standard porous tantalum cups plus cement were 18% cheaper than a single custom triflanged component, but 34% more expensive than a jumbo cup with bulk allograft. The technique is technically demanding: precise sequential reaming is required to avoid eccentric seating, and the surgeon must be prepared to convert to screw fixation if press-fit stability cannot be achieved. Formal hands-on training using 3D-printed bone models is recommended before clinical adoption.

Although press-fit provides initial mechanical stability, long-term construct survival depends on the biological behavior of both interfaces: trabecular metal/polymethylmethacrylate (PMMA) and PMMA/bone. The porous tantalum outer cup promotes osteoconduction; however, the interposed PMMA mantle remains largely bio-inert and may elicit a transient foreign-body reaction that peaks at 2–4 weeks, potentially impairing vascular ingrowth. Recent experimental work offers strategies to mitigate this problem. α-tricalcium phosphate (TCP)-doped PMMA shows clinically relevant loss of compressive strength beyond 3%, while β-TCP remains mechanically stable to 10%, suggesting β-TCP as the preferred variant for cement augmentation aimed at enhancing osteointegration without compromising implant fixation (21). In addition, introducing 2% hydroxyapatite of either 5 or 10 µm grain size transiently improves PMMA compressive strength, potentially extending implant longevity; however, higher concentrations offer no mechanical benefit and may jeopardize cement integrity under cyclic joint loading (22). Moreover, embedding fine glassy carbon (0.4–12 µm) into PMMA cement preserves load-bearing capacity up to 10%, whereas coarser particles (20–50 µm) precipitate a sharp decline in compressive strength above 5%, increasing the risk of early aseptic loosening after total joint replacement (23).

Several limitations inherent to the double-cup technique merit discussion. First, the requirement for at least three residual bony struts (ilium, ischium, pubis) to achieve press-fit stability may preclude its use in pelvic discontinuity or after extended periacetabular tumor resection. Second, despite the favorable frictional characteristics of trabecular metal, the absence of screws theoretically increases the risk of early migration if press-fit is suboptimal; radiostereometric analysis would be required to quantify micromotion in future series. Third, the inter-cup cement mantle introduces a potential interface for third-body wear. Although we observed no osteolysis at 22 months, longer follow-up is needed to monitor for polyethylene or cement debris. Lastly, patient-reported outcome measures (PROMs) were not obtained during the initial follow-up; however, PROMs have now been incorporated into our institutional revision hip protocol and will be systematically collected in future cases to allow more robust functional evaluation.

Although the 22-month improvement in HHS (39 to 78) is encouraging, we acknowledge that Paprosky type IIIB reconstructions must withstand ≥10 years of cyclic loading before durability can be assumed. Webb et al. reported 100% aseptic survival at 2.4 years, Zhang et al. described zero re-revisions at 5.1 years, and Chiarlone et al. documented 88.9% overall survival at 3 years (7, 8, 15). Nevertheless, progressive radiolucent lines or tantalum trabecular bone resorption may appear after 5 years, as seen in other revision constructs. We have therefore scheduled an annual clinical/radiographic review.

In conclusion, the use of the two-cup technique can be considered as an effective method for the treatment of Paprosky type III defects, achieving good initial stability at an early stage and allowing anatomical reconstruction of the lesion and restoration of the center of rotation of the hip with good clinical and imaging results.

## Data Availability

The original contributions presented in the study are included in the article/Supplementary Material, further inquiries can be directed to the corresponding author.
